# Cryopreservation of microglia enables single-cell RNA sequencing with minimal effects on disease-related gene expression patterns

**DOI:** 10.1016/j.isci.2021.102357

**Published:** 2021-03-25

**Authors:** Brenda Morsey, Meng Niu, Shetty Ravi Dyavar, Courtney V. Fletcher, Benjamin G. Lamberty, Katy Emanuel, Anna Fangmeier, Howard S. Fox

**Affiliations:** 1 Department of Neurological Sciences, University of Nebraska Medical Center, Omaha, NE 68198, USA; 2 Department of Genetics, Cell Biology and Anatomy; and Bioinformatics and Systems Biology Core, University of Nebraska Medical Center, Omaha, NE 68198, USA; 3 Antiviral Pharmacology Laboratory, UNMC Center for Drug Discovery, University of Nebraska Medical Center, Omaha, NE 68198, USA

**Keywords:** Molecular Neuroscience, Cellular Neuroscience, Transcriptomics

## Abstract

Microglia play a key role in brain development, normal homeostasis, and neurodegenerative disorders. Single-cell technologies have led to important findings about microglia, with many animal model studies using single-cell RNA sequencing (scRNA-seq), whereas most human specimen studies using archived frozen brains for single-nucleus RNA sequencing (snRNA-seq). However, microglia compose a small proportion of the total brain tissue; snRNAseq depletes expression of microglia activation genes that characterize many diseases. Here we examine the use of purified, cryopreserved microglia for scRNA-seq. Comparison of scRNA-seq on paired fresh and cryopreserved microglia from rhesus monkeys revealed a high level of correlation of gene expression between the two conditions. Disease-related genes were relatively unaffected, but an increase in immediate-early gene expression was present in cryopreserved cells. Regardless, changes in immediate-early gene expression are still detectable. Cryopreservation of microglia is a suitable procedure for prospectively archiving samples.

## Highlights

• High-quality scRNA-seq can be performed on cryopreserved microglia
• Cryopreservation of microglia does not affect disease-related gene expression
• Expression of immediate-early genes (IEG) is increased in cryopreserved microglia
• scRNA-seq of cyropreserved microglia reveals gene expression changes, including IEG

## Graphical abstract

**Figure:**
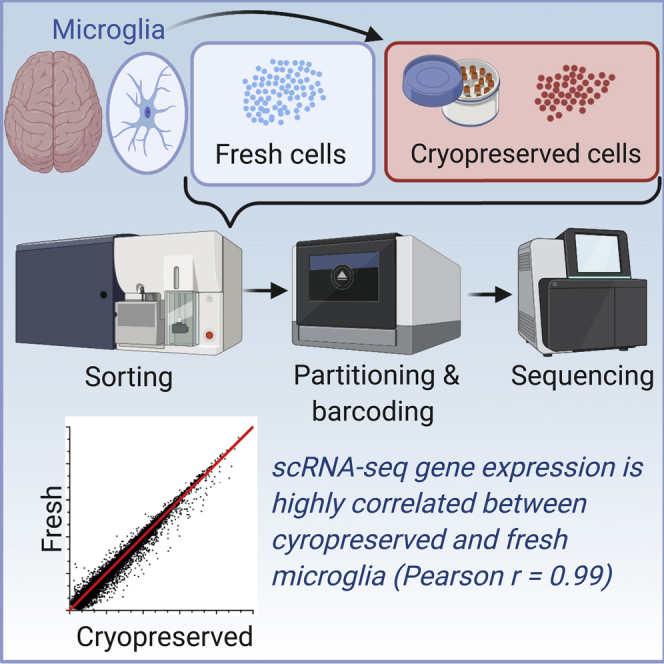

## Introduction

Microglia and central nervous system (CNS)-associated macrophages (CAM) make up the myeloid component of the CNS. In addition to their innate immune functions they are critical during brain development and for normal homeostasis. These cells also participate in the pathogenesis of CNS disorders, as well as protection and recovery from disease. The development and use of single-cell RNA sequencing (scRNA-seq) has led to significant advances in the study of many cell types, including microglia and CAM (Bonham et al., 2019; Geirsdottir et al., 2019; Gerrits et al., 2020; Goldmann et al., 2016; Hammond et al., 2019; Jordao et al., 2019; Keren-Shaul et al., 2017; Li et al., 2019; Masuda et al., 2019; Olah et al., 2018; Sousa et al., 2018; van der Poel et al., 2019). The study of microglia and CAM is complicated by their presence in the brain (and spinal cord), ruling out easy sampling such as for blood cells. Furthermore, in humans, access to biopsies and resections is quite limited.

Microglia and CAM originate from the yolk sac and populate the CNS during embryogenesis, whereas macrophages within the cerebrospinal fluid (CSF)-producing choroid plexus are derived from yolk sac precursors but are replaced during life by blood-derived cells (Goldmann et al., 2016). When fresh brain tissue is obtained, the tissue can be disassociated and cells purified for scRNA-seq; however, both these processes take time, and depending on when the tissue is procured (from experimental studies, the operating room, or the autopsy suite) and the specific protocol followed, it can be difficult to complete all processing in time to carry out the initial steps of scRNA-seq. Furthermore, microglia only make up approximately 10% of CNS cells (Lawson et al., 1992), limiting the number that can be studied.

To avoid the timing issue researchers have performed single nucleus RNA sequencing (snRNA-seq) using specimens stored frozen in tissue banks. For CNS studies, this technique has been frequently used to study specimens from those with Alzheimer disease (AD) (Del-Aguila et al., 2019; Grubman et al., 2019; Mathys et al., 2019; Zhou et al., 2020). Although archived samples still suffer from a low proportion of nuclei from microglial cells, the ability to use these archived samples represents a substantial advantage and may yield insight into disease pathogenesis. However, although snRNA-seq has been found to be applicable for many cell types, recently it was found to be not appropriate for the study of microglia. This is due to lack of detection of activation genes, which typify many disease states such as AD, and may help explain the inconsistency found between single-cell expression studies on AD (Thrupp et al., 2020).

A number of procedures have been used to preserve cells after isolation for future use in scRNA-seq, including methanol, paraformaldehyde, dithio-bis(succinimidyl propionate), and a commercial proprietary reagent (CellCover) fixation, as well as cryopreservation in dimethyl sulfoxide (DMSO) (Alles et al., 2017; Attar et al., 2018; Chen et al., 2018; Donlin et al., 2018; Gierahn et al., 2017; Guillaumet-Adkins et al., 2017; Thomsen et al., 2016; Zhou et al., 2020). A comparison of freshly isolated cells with cells cryopreserved in DMSO or fixed under two different conditions concluded that cryopreservation in DMSO was optimal for scRNA-seq (Wohnhaas et al., 2019). We have recently described the use of scRNA-seq on DMSO-cryopreserved microglia from Simian immunodeficiency virus (SIV)-infected rhesus monkeys, illuminating the effects of methamphetamine on SIV-induced CNS disease and modeling neuroHIV (in animals with SIV encephalitis [SIVE]) in those with and without a methamphetamine substance use disorder (Niu et al., 2020). Here we examined the effects of microglial cryopreservation on gene expression in microglia to determine whether this is a suitable method to archive cells for later scRNA-seq analysis or whether cryopreservation may also have an untoward effect on detecting activation as well as other disease-associated differentially expressed genes.

## Results

### Fresh and cryopreserved microglia yield proper metrics for scRNA-seq studies

**Figure 1:**
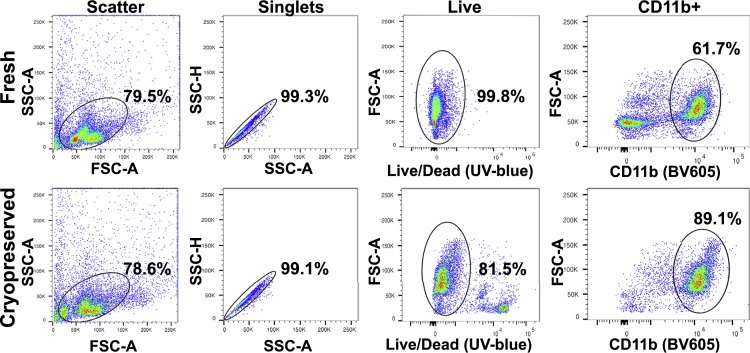
Sequence of flow cytometric purification of monkey microglia Forward and side scatter area (Scatter, far left) were used to select the cell population, followed by side scatter area and height to eliminate doublets (Singlets, second from left). Viable cells, determined by dye exclusion from live cells, were selected to eliminate dead cells (Live, third from left) followed by selection of CD11b-positive cells (far right). The proportion of cells selected in each gate within each panel is indicated, as are the fluorochromes for the two rightmost panels. Data from animal 86T are shown for the fresh (top) and cryopreserved (bottom) specimens. The fresh cells had a live, CD11b-negative population (top right panel), whereas these were not present in the cryopreserved cells, instead a population of dead cells was present (bottom, third panel from left). Both these populations mapped to the low FSC-A/SSC-A cells present in the gate shown in the top and bottom left panels.

We routinely isolate enriched populations of microglia and macrophages from the brains of nonhuman primates in our studies on SIV as a model for the neurological effects of HIV and cryopreserve them for use in our future experiments (Bortell et al., 2018; Chaudhuri et al., 2013; Marcondes et al., 2001; Watry et al., 1995). Recently we reported the use of these cryopreserved preparations in an scRNA-seq study (Niu et al., 2020). In order to investigate the effect of cryopreservation on scRNA-seq of microglia, we compared scRNA-seq results from freshly isolated and cryopreserved cells from two rhesus monkeys. These monkeys had been infected with SIV and treated with combination antiretroviral therapy, which suppressed the blood plasma virus to below the limit of detection. Fresh, enriched brain macrophages and microglia were isolated from two SIV-infected rhesus monkeys and then further purified (Figure 1, top), followed by capture on the 10x Genomics platform, and cDNA library production. Microglia were also cryopreserved using DMSO, and two months later, the cells were thawed and purified (Figure 1, bottom), captured on the 10x Genomics platform, and cDNA libraries were similarly synthesized.

cDNA libraries from both sets of cells were subjected to sequencing, and reads mapped to the rhesus genome. The scRNA-seq data from both animals and both conditions resulted in high-quality sequencing statistics (Table 1), notable for a high number of genes detected with little difference between the fresh and cryopreserved cells and low variability between the four samples. However, although equal numbers of cells were loaded for capture, an average of 33% fewer cells were captured in the cryopreserved samples compared with the fresh. The data were subjected to quality assurance and quality control followed by graph-based clustering and projection in three dimensions using Uniform Manifold Approximation and Projection (UMAP) (Figure S1). Examination of maker genes (those significantly overexpressed in each cluster) revealed a small cluster of lymphoid cells (likely cytotoxic T cells and/or natural killer cells, with marker genes including *CD3D*, *NKG7*, *GZMB*, and *LCK*), which were then excluded from further analysis. The remaining cells expressed typical markers of microglia, such as *P2RY12*, *CSF1R*, *AIF1*, and *CX3CR1* (Figure S1).

**Table 1:** Sequencing statistics

Sample	Estimated number of cells	Mean reads per cell	Median genes per cell	Sequencing saturation	Reads mapped confidently to genome	Fraction reads in cells	Total genes detected	Median UMI counts per cell
85T fresh	8,479	48,148	1,429	83.80%	86.30%	93.50%	19,535	2,551
86T fresh	6,338	64,263	1,928	72.50%	85.00%	93.30%	20,676	3,982
85T cryo	4,838	47,217	1,927	66.00%	86.70%	93.40%	19,864	3,772
86T cryo	4,921	45,556	1,727	72.30%	86.60%	94.10%	19,540	3,464

The scRNA-seq statistics of the samples from animals 85T and 86T, with both fresh and cryopreserved preparations. Data were generated by the Cell Ranger software of 10x Genomics.

### Gene expression patterns are closely correlated between cryopreserved and fresh microglia, including disease-associated genes

**Figure 2:**
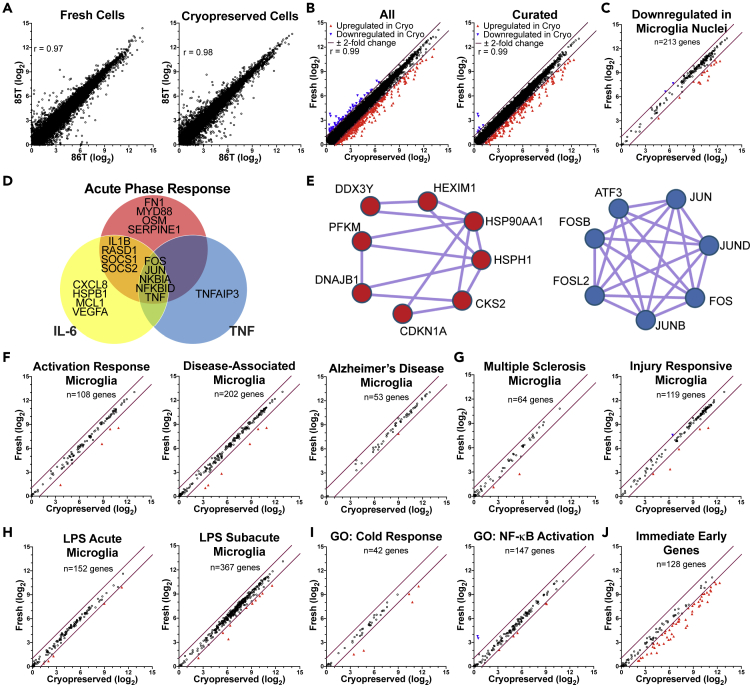
Evaluation of gene expression patterns between fresh and cryopreserved microglia, and comparison with other studies In the panels with dot plots, the purple diagonal lines indicate ±2-fold change, open black circles < |2|-fold change, red up-pointing triangles ≥2-fold increased, and blue down-pointing triangles ≥2-fold decreased, in the cryopreserved compared with fresh condition. (A) Dot plots comparing the expression of genes between the two monkeys in the fresh (left) and cryopreserved (right) conditions, revealing a high level of correlation using Pearson's r statistic (both p < 0.001). (B) Dot plots comparing the expression of genes between the two fresh and cryopreserved conditions, using all detected genes (left) and the curated protein-coding genes (right) as described in the text. Expression levels were highly correlated using Pearson's r statistic (both p < 0.001). (C) Expression of the genes decreased in snRNA-seq versus scRNA-seq from human microglia (Thrupp et al., 2020), in the monkey microglia fresh and cryopreserved conditions. (D) Venn diagram indicating overlapping DEGs found in the indicated IPA signaling pathways. (E) MCODE interactome networks of DEGs revealing networks enriched for the cellular response to heat pathway (left), and the AP1 pathway (right). (F–J) Expression of genes in the indicated lists from microglia scRNA-seq and snRNA-seq studies, as well as Gene Ontology categories and the curated IEGs in the fresh and cryopreserved monkey microglia.

We then explored single microglial cell gene expression from the two animals under the fresh and cryopreserved conditions. The gene expression levels from the different monkeys were highly correlated from both the fresh and cryopreserved conditions (Figure 2A). In comparing the cryopreserved with the fresh cells, a low proportion showed a 2-fold or greater change (all with a false discover rate <0.05), with 2.0% increased and 0.4% decreased in the cryopreserved cells compared with the fresh cells (Figure 2B). We then curated the gene list to those that are present in genomic DNA (as opposed to mitochondrial DNA), encoded proteins (versus regulatory RNAs), and excluded those encoding ribosomal proteins. A high level of correlation was again seen within the curated list, with only 1.8% of the gene expression values increased, and 0.1% decreased, in the cryopreserved cells compared with the fresh cells (Figure 2B).

snRNA-seq, which has been a key method in the analysis of brain cells and other cells from frozen archived specimens, has been found to be inappropriate for assessment of human microglia due to loss of ability to assess expression of key genes involved in disease pathogenesis when compared with scRNA-seq (Thrupp et al., 2020). As both monkeys and humans are primates, we assessed our findings on the effect of cryopreservation on microglial scRNA-seq in relation to that study. Using the criteria of expression change of 2-fold or greater change (as used by Thrupp et al., 2020), we found that 8.0% of the genes that were underrepresented in nuclei in snRNA-seq were increased in the cryopreserved compared with fresh cells we analyzed by scRNA-seq, whereas 0.9% were decreased (Figure 2C).

To obtain functional information on the expression changes resulting from cryopreservation, we analyzed a list of differentially expressed genes (DEGs) (538 genes showing a fold change of >|1.5| and false discovery rate of <0.05, Table S1) between the fresh and cryopreserved cells using Ingenuity Pathway Analysis (IPA). This revealed canonical pathway alterations (Table S2) including three signaling pathways: acute phase response, IL6, and TNFR2, which contained overlapping DEGs (Figure 2D). We next submitted the DEGs to Metascape to perform interactome analysis (using MCODE), which revealed densely connected protein networks, and subsequently performed gene set enrichment analysis. One network (Figure 2E, left) was highly enriched for cellular response to heat (gene ontology pathway 0034605, p = 10^−7.6^), and a second (Figure 2E, right) was highly enriched for the AP1 pathway (protein interaction database pathway M167, p = 10^−16.4^). The AP1 pathway is activated as part of the acute phase response (Hattori et al., 1993), linking these two independent analyses.

In the snRNA-seq versus scRNA-seq comparison (Thrupp et al., 2020), the genes that were underrepresented in nuclei were highly represented in lists of genes whose expressions were increased in AD. Two of these lists were from scRNA-seq AD model studies in mice, denoting activation response microglia (ARM) and disease-associated microglia (DAM) (Keren-Shaul et al., 2017; Sala Frigerio et al., 2019), and a third is from a snRNA-seq study of human AD (Mathys et al., 2019). This led to the conclusion that snRNA-seq was not appropriate for the study of microglia. We found that only a small proportion of the genes listed had their expression changed by cryopreservation, with only 3.7% of the ARM, 3.0% of DAM, and 1.9% of the AD genes showing increased expression in cryopreserved cells; none showed decreased expression (Figure 2F).

We next evaluated our data using other relevant microglial gene lists (Table S3). Of the altered genes in an snRNA-seq examination of a neuroinflammatory demyelinating disease, human multiple sclerosis (MS) (Schirmer et al., 2019), the cryopreserved cells had 3.1% increased, and none decreased, gene expression. Furthermore, in a mouse scRNA-seq study of response to a demyelinating injury (denoted injury response microglia) caused by lysolecithin injection into the brain (Hammond et al., 2019), 4.2% of the genes found to have change were increased, and 0.8% decreased, in our cryopreserved microglia relative to fresh cells (Figure 2G).

Inflammatory conditions were examined in scRNA-seq studies in mice, using microglia isolated 3 (acute) or 24 h (subacute) after an intraperitoneal injection of lipopolysaccharide (LPS) (Gerrits et al., 2020; Sousa et al., 2018). It was found that 2.6% and 3.0%, respectively, of the genes with increased expression were also increased in cryopreserved versus fresh microglia. None of the genes in the LPS gene lists showed decreased expression in the cryopreserved cells relative to the fresh cells in our study (Figure 2H).

As the cells were subject to cold, we also examined genes responsive to cold stress (using Gene Ontology GO: 0009409, response to cold, Table S3). Although a relatively small number of genes (42) are in this set, five (12%) of the genes were increased in the cryopreserved microglia relative to the fresh cells; none were decreased. Interestingly, cold stress in mice has been found to activate the nuclear factor (NF)-κB signaling pathway in microglia (Xu et al., 2020). Therefore, we examined genes known to be involved in activation of this pathway (using Gene Ontology GO:0051092, positive regulation of NF-κB transcription factor activity, Table S3). Here 3.4% were upregulated, and 1.4% downregulated, in cryopreserved microglia versus fresh cells (Figure 2I).

### Immediate-early genes are increased in cryopreserved microglia

Previous studies revealed that induction of immediate-early genes (IEGs) can occur due to tissue processing for scRNA-seq (van den Brink et al., 2017), with microglia among the most prominent IEG responders (Wu et al., 2017). IEGs are genes whose transcripts are rapidly expressed after cellular stimulation, without the need for new protein synthesis to control this expression (Herschman, 1991). Using a curated list of IEGs (Wu et al., 2017) (Table S3), we found a high level of change in expression of these genes, with 55 of 128 (43%) homologous to the IEG list showing a greater than 2-fold increase in the cryopreserved cells relative to the fresh cells and none exhibiting a decreased expression (Figure 2J). This finding is consistent with the elevations in acute phase response and AP1 pathways (Figures 2D and 2E), as IEGs precede activation of these pathways.

In order to examine the statistical significance of changes in gene expression, we used gene set enrichment analysis (GSEA) to determine if one or more of the gene sets is significantly enriched or depleted in expression in the cryopreserved versus fresh cells. The 11 gene sets were assessed using the complete expression list. Although all show some degree of enrichment in the cryopreserved cells, the IEGs have the highest level, and only the IEGs were significantly enriched in expression (Table 2).

**Table 2:** Gene set enrichment analysis

Gene list	Size	NES	NOM p-val	FDR q-val
Immediate-early genes	128	1.984	0.0	0.0
GO: Response to cold	42	1.188	0.145	0.319
Multiple sclerosis microglia	64	1.101	0.243	0.702
Down-regulated in microglia nuclei	213	1.029	0.377	1.0
GO: Positive regulation of NF-κB	147	0.956	0.654	1.0
Acute LPS microglia	152	0.941	0.708	1.0
Activation response microglia	108	0.935	0.679	1.0
Injury response microglia	119	0.830	0.923	1.0
Disease-associated microglia	202	0.817	0.966	1.0
Alzheimer disease microglia	53	0.701	0.966	1.0
Subacute LPS microglia	367	0.680	1.0	0.992

The gene sets are the same as those assessed in Figure 2. The size is the number of genes within each gene set for which expression was measured in the current experiment. The normalized enrichment score (NEG) indicates the degree to which a gene set is overrepresented for differences in gene set size, normalized for correlations between gene sets and the expression dataset. The positive numbers connote enrichment in the cryopreserved microglia. The nominal (NOM) p value reflects the significance of single set, whereas the false discovery rate (FDR) q-value adjusts the significance value for gene set size and multiple hypotheses testing.

### Changes in IEGs can still be assessed in cryopreserved microglia

**Figure 3:**
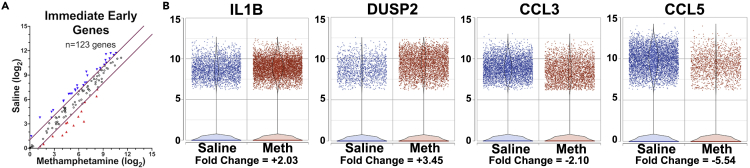
IEGs can change in expression between conditions in cryopreserved microglia (A) Dot plots of IEGs expression in monkeys with SIVE treated with saline or methamphetamine; symbols as in Figure 2. (B) Overlaid dot and violin plots indicate expression levels of the selected IEGs in the two conditions, with fold change of the methamphetamine relative to the saline condition indicated. All data from (Niu et al., 2020).

Despite an increase in expression of IEGs due to cryopreservation, they still may be responsive to changes in experimental conditions. To assess this possibility, we examined IEG expression in our prior study of the effect of drug abuse on a model of neuroHIV in monkeys, which utilized cryopreserved microglia for scRNA-seq (Niu et al., 2020). In fact, a number of the IEGs were altered between the saline and methamphetamine conditions, with some IEGs increased (e.g., *IL1B* and *DUSP2*), and others decreased (e.g., *CCL3* and *CCL5*), in expression by methamphetamine compared with saline treatment of the animals (Figure 3). Thus, there is no ceiling effect or blanket inability to detect differences of IEGs in response to different physiological conditions.

### Trajectory analysis reveals change in state in the cryopreserved microglia

**Figure 4:**
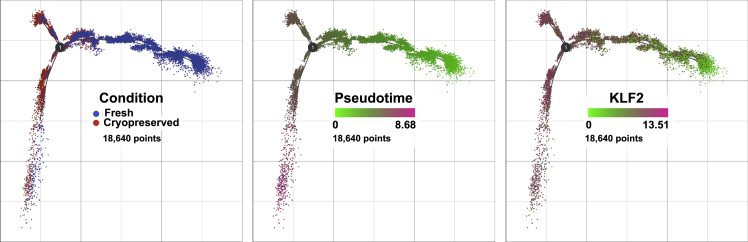
Trajectory analysis Trajectory analysis of cells from the fresh and cryopreserved conditions. Cells are color-coded by condition (left), calculated pseudotime (center), and expression of the IEG *KLF2* (right)

To understand the cells' responses to the cryopreservation and subsequent recovery in scRNA-seq experiments, we conducted trajectory inference followed by pseudotime analysis on the monkey microglia. Trajectory analysis enables investigation of changes as cells progress through a dynamic process, here from fresh cells to cryopreserved, examining the transition from one condition to the other. This is then displayed as a pseudotime graph, with values corresponding to progress along the change between conditions. As there were different numbers of cells in the samples, to avoid bias between conditions the cell number was down-sampled in each to the amount in the smallest of the four samples (4,660 cells). To facilitate the analysis, the identified DEGs (Table S1) were used. As shown in Figure 4 (left and middle), the cells begin the trajectory from the presumed zero pseudotime point on the right. The vast majority (95.5%) of the fresh cells, as well 34.5% of the cryopreserved cells, lie in the initial stage. The trajectory then diverges into two separate paths at “1” with an upward-facing group containing 2.2% of the fresh cells and 40.1% of the cryopreserved cells and a downward-facing group containing 3.3% of the fresh cells and 25.3% of the cryopreserved cells. An example of the expression pattern of DEG is shown for the IEGs *KLF2* (Figure 4, right) as well as *FOS, PER1*, and *CCL3* (Figure S2). No one gene appears to drive the transition between fresh and cryopreserved; rather, as in other stressors, many of the IEGs show increased expression.

### Analysis of tissue macrophage scRNA-seq data reveals IEGs are also increased by cryopreservation

**Figure 5:**
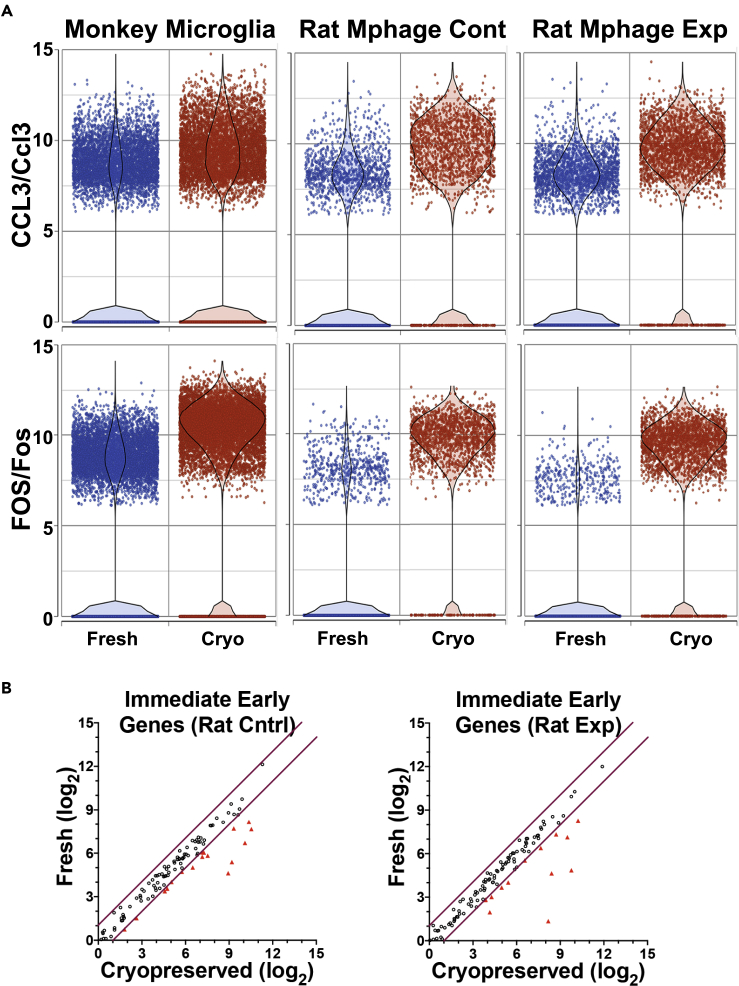
Expression of selected IEGs in monkey microglia and rat macrophages (A) Overlaid dot and violin plots indicate expression levels of the shown genes in cells from the current monkey microglia experiment (left) and the rat liver macrophages (control diet, center, and experimental diet, right), revealing increased expression in the cryopreserved compared with fresh condition. (B) Dot plots of IEGs expression in rat liver macrophages in the control (left) and experimental diet (right) conditions; symbols as in Figure 2. Rat liver macrophage data from (Wohnhaas et al., 2019).

A recent study compared freshly isolated cells with cryopreserved cells under different conditions and concluded that cryopreservation in DMSO, as performed here, was optimal (Wohnhaas et al., 2019). As part of this study, immune cells isolated from the livers of rats subjected to a choline-deficient diet (experimental) were compared with those fed a control diet (two rats per condition) using scRNA-seq on the 10x Genomics platform, again as performed here. To assess whether the effects of cryopreservation on IEGs are specific to microglia or present in other cell types, we downloaded the data from GEO (Series GSE127248) and filtered for the macrophages using graph-based clustering and marker genes (Figure S3). Combining the lists of differentially expressed genes found in either the experimental or control conditions between fresh and DMSO cryopreserved cells revealed a low proportion of greater than 2-fold changes, 1.34% in the control condition and 0.91% in the experimental. Interestingly, between the two conditions in the rat macrophage experiment, only 16 genes were in common regarding increased expression due to cryopreservation. Thirteen (81%) of these are IEGs. These include the expression of the IEGs *CCL3/Ccl3* (3.49-fold in monkey microglia, 4.59-fold and 3.89-fold in control and experimental rat liver macrophages, respectively) and *FOS/Fos* (6.51-, 10.45-, and 30.09-fold) (Figure 5A). Examining the list of curated IEGs, 16.2% of the genes showed increased expression in the cryopreserved cells from rats fed the control diet, whereas 13.0% were increased in those fed the experimental diet (Figure 5B). Trajectory analysis revealed that the rat liver macrophages also showed a progression from the fresh to cryopreserved cells, albeit with a more complex branching pattern (Figure S4).

## Discussion

The analysis of microglia by single-cell technologies has led to an increased understanding of various types of microglia in development, homeostasis, and changes due to aging and disease. Such studies have yielded important clues into the function of microglia in these processes. Single-cell transcriptomic studies from human specimens are often done with snRNA-seq; however expression of disease-related genes can be lost (Thrupp et al., 2020). Here we find that isolation and cryopreservation of microglia can be performed, and the cells can then be archived for later purification and use in scRNA-seq, with minimal changes in expression of disease-related genes. In both our study and a prior study on macrophages in rats (Wohnhaas et al., 2019), an increase in expression of IEGs was found in cryopreserved cells. This may be, in part, due to the freezing and/or recovery process used in cryopreservation, as previous studies have noted that IEGs are upregulated during tissue processing for scRNA-seq, especially notable in microglia (Wu et al., 2017).

IEGs were initially characterized as rapidly transcribed genes induced after growth factor stimulation of cells (Lau and Nathans, 1987). They are also induced in response to cellular differentiation as well as inflammation, neuronal activity, and stress (Bahrami and Drablos, 2016). Although we did not see enrichment of expression of genes associated with the response to cold, the IEG induction is likely due to cellular stress in the freezing and/or recovery process and has been noted by others in cryopreserved cells (Ruf-Zamojski et al., 2018).

Although IEGs are altered in the cryopreserved cells, this does not mean that these genes are not informative in studies. First, despite all precautions, isolation procedures are prone to lead to a level of IEG activation. Differences in IEG expression using cryopreserved microglia in scRNA-seq can still be detected between different *in vivo* conditions (Figure 3). Furthermore, when considering the genes whose expressions were suppressed in snRNA-seq relative to scRNA-seq (Thrupp et al., 2020), many of these were still significantly altered in studies on AD and MS (Mathys et al., 2019; Schirmer et al., 2019) (see Table S3). It is not clear which method best represents the status *in vivo*, as all have caveats. If one is not comparing results obtained by the same method, means of accounting for the known differences should be used.

Archiving of isolated microglia can lead to their productive use in subsequent studies without the loss of disease-related information. In addition, such cells can be used in a variety of additional studies, including other emerging single-cell technologies or long-established ones such as flow cytometry, in addition to nucleic acid, protein, or small molecule analyses. Furthermore, as cells are viable when recovered, cell culture experiments are also made possible. Cryopreserving cells will also allow the choice of specimens for future studies, instead of having to process for scRNA-seq before one knows the true utility of the specimen, such as not knowing the true diagnosis pending neuropathological or other evaluation. In addition to microglia, we have also reported the successful use of cryopreserved matured monocytes using scRNA-seq (Leon-Rivera et al., 2020). Other groups have reported the isolation and use of microglia from fresh autopsy human brain tissue from brain banks, in particular for studies on AD, and many have utilized microglia from fetal tissue as well as surgical resections for intractable epilepsy (Lue et al., 2019). We show here that cryopreserved microglia can be utilized for scRNA-seq in addition to other known uses.

Knowledge of the role of microglia in CNS function and dysfunction continues to grow, and single-cell technologies have contributed to this growth. In addition, techniques to perform single-cell procedure and analyses continue to expand. Cryopreservation will enable valuable specimens to be used for current and future studies to elucidate the role of microglia in CNS function and dysfunction.

### Limitations of this study

Limitations include the use of only two biological replicates, and the examination on rhesus monkey microglia, as opposed to specimens from humans or other animal species. Furthermore, we only examined SIV-infected animals under suppressive antiretroviral treatment, and not those with other diseases or control animals. We do note the replicates were closely correlated, and that, among the different species examined by scRNA-seq, rhesus monkey microglia were found to show the highest similarity to human microglia (Geirsdottir et al., 2019). It has been found that performing tissue disruption and cellular isolation procedures in the cold was found to reduce or eliminate IEG upregulation (Adam et al., 2017; Wu et al., 2017). However, others have found that performing brain dissociation in the cold results in poor yield and lower viability and that the cells are prone to clump (Bordt et al., 2020). The reason for the decreased number of cryopreserved, relative to fresh, cells captured in the processing for scRNA-seq is not known, but may represent increased fragility of the cryopreserved cells. Cellular damage in cryopreserved cells used for scRNA-seq has been previously reported (Guillaumet-Adkins et al., 2017). Whether cryopreservation may introduce a bias in the cells analyzed, as with any isolation and purification procedure, remains to be determined, and future studies are needed to assess such a potential bias. We have successfully performed scRNA-seq from microglia samples that have been cryopreserved for over 7 years (Niu et al., 2020). Future studies can also address any limits on storage time on cryopreserved microglia, and changes that may occur due to different storage times. It would also be of interest if a similar study on brains frozen for different times was performed. Finally, direct comparison of different procedures (snRNA-seq, scRNA-seq from fresh and cryopreserved cells) can assist in comparing the methods and enabling meta-analyses to be done from studies performed with the different platforms.

### Resource availability

#### Lead contact

Howard Fox, University of Nebraska Medical Center, hfox@unmc.edu.

#### Materials availability

This study did not generate new unique reagents.

#### Data and code availability

The scRNA-seq data from this study has been deposited in the NCBI GEO database, accession # GSE162663.

## Methods

All methods can be found in the accompanying transparent methods supplemental file.

## Supplemental information

Document S1. Transparent methods and Figures S1–S4

Table S1. Differentially expressed genes, related to figures 2 and 4 The 538 DEGs, showing a fold change of >|1.5| and false discovery rate of <0.05, are listed. The gene symbol identifier (Gene ID), the total number of counts detected (Total Counts), the values for the comparisons between conditions (cryopreserved versus fresh), the false discovery rate (FDR), ratio, and fold change are given. The least square mean (LSMean) of the normalized expression value for each gene cryopreserved and fresh cells is listed.

Table S2. Pathway analysis, related to figure 2 IPA canonical pathways showing those with significant (FDR q-value, Benjamini and Hochberg false discover rate, < 0.05) activation (*Z* score ≥ 1), as determined by the differentially expressed genes between cryopreserved and fresh cells.

Table S3. Gene sets, related to figure 2, table 2, and discussion The 11 gene sets assessed are listed. The original list (Original), and those with homologs in the rhesus monkey genome (Mapped, with the rhesus gene ID and human gene ID) are indicated. Gene set lists were obtained from Table S1 in (Thrupp et al., 2020), and the other sources referenced in the results.

## References

[bib1] Adam M., Potter A.S., Potter S.S. (2017). Psychrophilic proteases dramatically reduce single-cell RNA-seq artifacts: a molecular atlas of kidney development. Development.

[bib2] Alles J., Karaiskos N., Praktiknjo S.D., Grosswendt S., Wahle P., Ruffault P.L., Ayoub S., Schreyer L., Boltengagen A., Birchmeier C. (2017). Cell fixation and preservation for droplet-based single-cell transcriptomics. BMC Biol..

[bib3] Attar M., Sharma E., Li S., Bryer C., Cubitt L., Broxholme J., Lockstone H., Kinchen J., Simmons A., Piazza P. (2018). A practical solution for preserving single cells for RNA sequencing. Sci. Rep..

[bib4] Bahrami S., Drablos F. (2016). Gene regulation in the immediate-early response process. Adv. Biol. Regul..

[bib5] Bonham L.W., Sirkis D.W., Yokoyama J.S. (2019). The transcriptional landscape of microglial genes in aging and neurodegenerative disease. Front. Immunol..

[bib6] Bordt E.A., Block C.L., Petrozziello T., Sadri-Vakili G., Smith C.J., Edlow A.G., Bilbo S.D. (2020). Isolation of microglia from mouse or human tissue. STAR Protoc..

[bib7] Bortell N., Basova L., Najera J.A., Morsey B., Fox H.S., Marcondes M.C.G. (2018). Sirtuin 1-chromatin-binding dynamics points to a common mechanism regulating inflammatory targets in SIV infection and in the aging brain. J. Neuroimmune Pharmacol..

[bib8] Chaudhuri A.D., Yelamanchili S.V., Marcondes M.C., Fox H.S. (2013). Up-regulation of microRNA-142 in simian immunodeficiency virus encephalitis leads to repression of sirtuin1. FASEB J..

[bib9] Chen J., Cheung F., Shi R., Zhou H., Lu W., Consortium C.H.I. (2018). PBMC fixation and processing for Chromium single-cell RNA sequencing. J. Transl. Med..

[bib10] Del-Aguila J.L., Li Z., Dube U., Mihindukulasuriya K.A., Budde J.P., Fernandez M.V., Ibanez L., Bradley J., Wang F., Bergmann K. (2019). A single-nuclei RNA sequencing study of Mendelian and sporadic AD in the human brain. Alzheimers Res. Ther..

[bib11] Donlin L.T., Rao D.A., Wei K., Slowikowski K., McGeachy M.J., Turner J.D., Meednu N., Mizoguchi F., Gutierrez-Arcelus M., Lieb D.J. (2018). Methods for high-dimensional analysis of cells dissociated from cryopreserved synovial tissue. Arthritis Res. Ther..

[bib12] Geirsdottir L., David E., Keren-Shaul H., Weiner A., Bohlen S.C., Neuber J., Balic A., Giladi A., Sheban F., Dutertre C.A. (2019). Cross-species single-cell analysis reveals divergence of the primate microglia program. Cell.

[bib13] Gerrits E., Heng Y., Boddeke E., Eggen B.J.L. (2020). Transcriptional profiling of microglia; current state of the art and future perspectives. Glia.

[bib14] Gierahn T.M., Wadsworth M.H., Hughes T.K., Bryson B.D., Butler A., Satija R., Fortune S., Love J.C., Shalek A.K. (2017). Seq-Well: portable, low-cost RNA sequencing of single cells at high throughput. Nat. Methods.

[bib15] Goldmann T., Wieghofer P., Jordao M.J., Prutek F., Hagemeyer N., Frenzel K., Amann L., Staszewski O., Kierdorf K., Krueger M. (2016). Origin, fate and dynamics of macrophages at central nervous system interfaces. Nat. Immunol..

[bib16] Grubman A., Chew G., Ouyang J.F., Sun G., Choo X.Y., McLean C., Simmons R.K., Buckberry S., Vargas-Landin D.B., Poppe D. (2019). A single-cell atlas of entorhinal cortex from individuals with Alzheimer's disease reveals cell-type-specific gene expression regulation. Nat. Neurosci..

[bib17] Guillaumet-Adkins A., Rodriguez-Esteban G., Mereu E., Mendez-Lago M., Jaitin D.A., Villanueva A., Vidal A., Martinez-Marti A., Felip E., Vivancos A. (2017). Single-cell transcriptome conservation in cryopreserved cells and tissues. Genome Biol..

[bib18] Hammond T.R., Dufort C., Dissing-Olesen L., Giera S., Young A., Wysoker A., Walker A.J., Gergits F., Segel M., Nemesh J. (2019). Single-cell RNA sequencing of microglia throughout the mouse lifespan and in the injured brain reveals complex cell-state changes. Immunity.

[bib19] Hattori M., Tugores A., Westwick J.K., Veloz L., Leffert H.L., Karin M., Brenner D.A. (1993). Activation of activating protein 1 during hepatic acute phase response. Am. J. Physiol..

[bib20] Herschman H.R. (1991). Primary response genes induced by growth factors and tumor promoters. Annu. Rev. Biochem..

[bib21] Jordao M.J.C., Sankowski R., Brendecke S.M., Sagar, Locatelli G., Tai Y.H., Tay T.L., Schramm E., Armbruster S., Hagemeyer N. (2019). Single-cell profiling identifies myeloid cell subsets with distinct fates during neuroinflammation. Science.

[bib22] Keren-Shaul H., Spinrad A., Weiner A., Matcovitch-Natan O., Dvir-Szternfeld R., Ulland T.K., David E., Baruch K., Lara-Astaiso D., Toth B. (2017). A unique microglia type Associated with restricting development of alzheimer's disease. Cell.

[bib23] Lau L.F., Nathans D. (1987). Expression of a set of growth-related immediate early genes in BALB/c 3T3 cells: coordinate regulation with c-fos or c-myc. Proc. Natl. Acad. Sci. U S A.

[bib24] Lawson L.J., Perry V.H., Gordon S. (1992). Turnover of resident microglia in the normal adult mouse brain. Neuroscience.

[bib25] Leon-Rivera R., Morsey B., Niu M., Fox H.S., Berman J.W. (2020). Interactions of monocytes, HIV, and ART identified by an innovative scRNAseq pipeline: pathways to reservoirs and HIV-associated comorbidities. mBio.

[bib26] Li Q., Cheng Z., Zhou L., Darmanis S., Neff N.F., Okamoto J., Gulati G., Bennett M.L., Sun L.O., Clarke L.E. (2019). Developmental heterogeneity of microglia and brain myeloid cells revealed by deep single-cell RNA sequencing. Neuron.

[bib27] Lue L.F., Beach T.G., Walker D.G. (2019). Alzheimer's disease Research using human microglia. Cells.

[bib28] Marcondes M.C., Burudi E.M., Huitron-Resendiz S., Sanchez-Alavez M., Watry D., Zandonatti M., Henriksen S.J., Fox H.S. (2001). Highly activated CD8(+) T cells in the brain correlate with early central nervous system dysfunction in simian immunodeficiency virus infection. J. Immunol..

[bib29] Masuda T., Sankowski R., Staszewski O., Bottcher C., Amann L., Sagar, Scheiwe C., Nessler S., Kunz P., van Loo G. (2019). Spatial and temporal heterogeneity of mouse and human microglia at single-cell resolution. Nature.

[bib30] Mathys H., Davila-Velderrain J., Peng Z., Gao F., Mohammadi S., Young J.Z., Menon M., He L., Abdurrob F., Jiang X. (2019). Single-cell transcriptomic analysis of Alzheimer's disease. Nature.

[bib31] Niu M., Morsey B., Lamberty B.G., Emanuel K., Yu F., Leon-Rivera R., Berman J.W., Gaskill P.J., Matt S.M., Ciborowski P.S. (2020). Methamphetamine increases the proportion of SIV-infected microglia/macrophages, alters metabolic pathways, and elevates cell death pathways: a single-cell analysis. Viruses.

[bib32] Olah M., Patrick E., Villani A.C., Xu J., White C.C., Ryan K.J., Piehowski P., Kapasi A., Nejad P., Cimpean M. (2018). A transcriptomic atlas of aged human microglia. Nat. Commun..

[bib33] Ruf-Zamojski F., Ge Y., Nair V., Zamojski M., Pincas H., Toufaily C., Tome-Garcia J., Stoeckius M., Stephenson W., Smith G.R. (2018). Single-cell stabilization method identifies gonadotrope transcriptional dynamics and pituitary cell type heterogeneity. Nucleic Acids Res..

[bib34] Sala Frigerio C., Wolfs L., Fattorelli N., Thrupp N., Voytyuk I., Schmidt I., Mancuso R., Chen W.T., Woodbury M.E., Srivastava G. (2019). The major risk factors for alzheimer's disease: age, sex, and genes modulate the microglia response to abeta plaques. Cell Rep..

[bib35] Schirmer L., Velmeshev D., Holmqvist S., Kaufmann M., Werneburg S., Jung D., Vistnes S., Stockley J.H., Young A., Steindel M. (2019). Neuronal vulnerability and multilineage diversity in multiple sclerosis. Nature.

[bib36] Sousa C., Golebiewska A., Poovathingal S.K., Kaoma T., Pires-Afonso Y., Martina S., Coowar D., Azuaje F., Skupin A., Balling R. (2018). Single-cell transcriptomics reveals distinct inflammation-induced microglia signatures. EMBO Rep..

[bib37] Thomsen E.R., Mich J.K., Yao Z., Hodge R.D., Doyle A.M., Jang S., Shehata S.I., Nelson A.M., Shapovalova N.V., Levi B.P. (2016). Fixed single-cell transcriptomic characterization of human radial glial diversity. Nat. Methods.

[bib38] Thrupp N., Sala Frigerio C., Wolfs L., Skene N.G., Fattorelli N., Poovathingal S., Fourne Y., Matthews P.M., Theys T., Mancuso R. (2020). Single-nucleus RNA-seq is not suitable for detection of microglial activation genes in humans. Cell Rep..

[bib39] van den Brink S.C., Sage F., Vertesy A., Spanjaard B., Peterson-Maduro J., Baron C.S., Robin C., van Oudenaarden A. (2017). Single-cell sequencing reveals dissociation-induced gene expression in tissue subpopulations. Nat. Methods.

[bib40] van der Poel M., Ulas T., Mizee M.R., Hsiao C.C., Miedema S.S.M., Adelia, Schuurman K.G., Helder B., Tas S.W., Schultze J.L. (2019). Transcriptional profiling of human microglia reveals grey-white matter heterogeneity and multiple sclerosis-associated changes. Nat. Commun..

[bib41] Watry D., Lane T.E., Streb M., Fox H.S. (1995). Transfer of neuropathogenic simian immunodeficiency virus with naturally infected microglia. Am. J. Pathol..

[bib42] Wohnhaas C.T., Leparc G.G., Fernandez-Albert F., Kind D., Gantner F., Viollet C., Hildebrandt T., Baum P. (2019). DMSO cryopreservation is the method of choice to preserve cells for droplet-based single-cell RNA sequencing. Sci. Rep..

[bib43] Wu Y.E., Pan L., Zuo Y., Li X., Hong W. (2017). Detecting activated cell populations using single-cell RNA-seq. Neuron.

[bib44] Xu B., Lang L.M., Lian S., Guo J.R., Wang J.F., Liu J., Yang H.M., Li S.Z. (2020). Neuroinflammation induced by secretion of acetylated HMGB1 from activated microglia in hippocampi of mice following chronic cold exposure. Brain Res..

[bib45] Zhou Y., Song W.M., Andhey P.S., Swain A., Levy T., Miller K.R., Poliani P.L., Cominelli M., Grover S., Gilfillan S. (2020). Human and mouse single-nucleus transcriptomics reveal TREM2-dependent and TREM2-independent cellular responses in Alzheimer's disease. Nat. Med..

