# Supplementary material for: Cryopreservation of microglia enables single-cell RNA sequencing with minimal effects on disease-related gene expression patterns
Source: iScience. 2021 Mar 25;24(4):102357. doi: 10.1016/j.isci.2021.102357 (PMC8044433; doi:10.1016/j.isci.2021.102357)
Supplement: Document S1. Transparent methods and Figures S1–S4 [file mmc1.pdf]

iScience, Volume *24*

Supplemental information

Cryopreservation of microglia enables single-cell RNA sequencing with minimal effects on disease-related gene expression patterns

Brenda Morsey, Meng Niu, Shetty Ravi Dyavar, Courtney V. Fletcher, Benjamin G. Lamberty, Katy Emanuel, Anna Fangmeier, and Howard S. Fox

Supplemental Figures

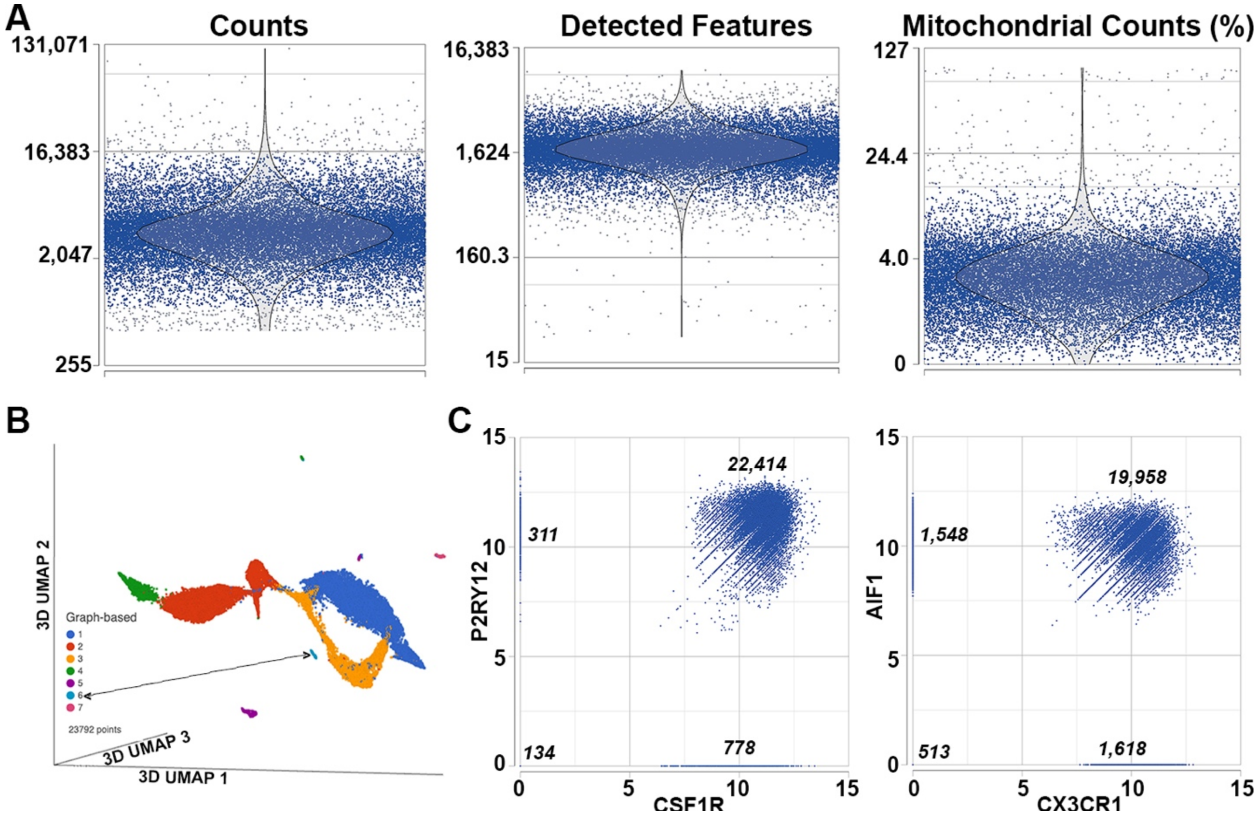

Figure S1. Quality assurance and quality control (QA/QC) of monkey microglia (Related to Table 1 and Results). QA/QC was performed using overlaid dot and violin plots (with the Y-axis in log-scale) of the indicated measures. **(A)** Counts were filtered to include cells with between 800-15,000 total counts (left), Detected Features to include cells with between 300-5,000 genes detected (middle), and the percent of counts mapping to the Mitochondrial genome filtered to cells with 15% or less (right). **(B)** To remove non-myeloid cells, graph-based clustering was performed followed by projection in three dimensions using UMAP. Cluster 6 (double-headed arrow) was enriched in cells expressing genes found in lymphocytes, specifically cytotoxic T cells and/or natural killer cells, and cells in this cluster were excluded from further analysis. **(C)** Dot plots showing expression of microglia marker genes from the 23,637 cells remaining cells after elimination of cluster 6. The number of cells expressing both indicated markers is indicated in the upper right, those with only one of the markers at the upper left and right (along axes), and neither marker lower left (near origin).

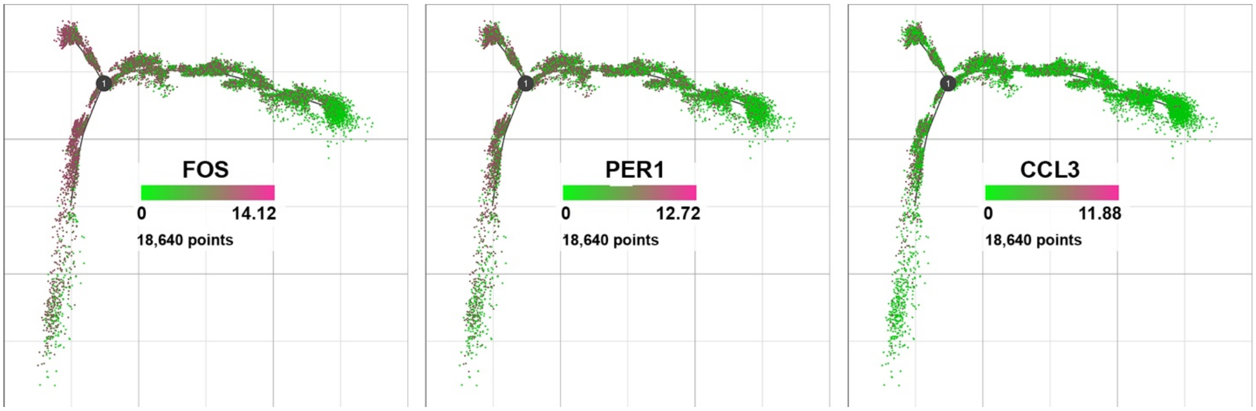

Figure S2. IEG expression along trajectory analysis (Related to Figure 4). Trajectory analysis of the fresh to cryopreserved state change, with cells color-coded by expression of the indicated IEG.

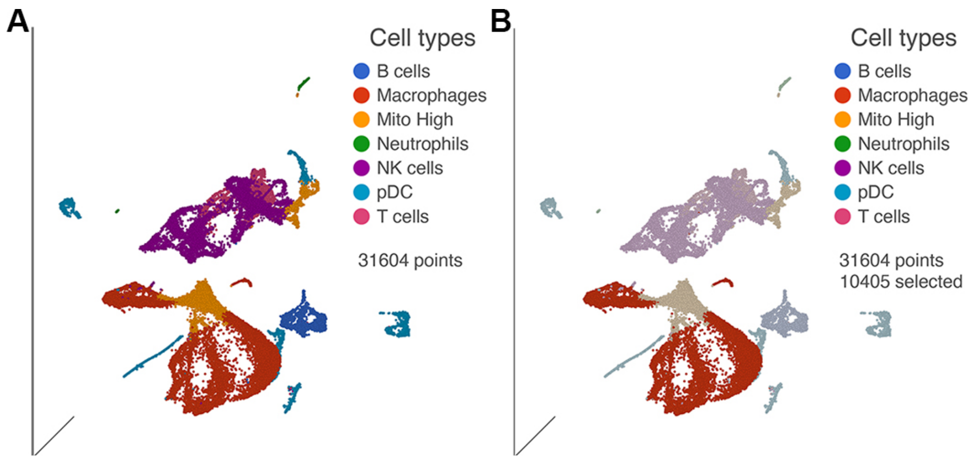

Figure S3. **Identification of macrophages in the rat liver immune cell experiment (Related to Figure 5). (A)** Graph-based clustering revealed groups of cells with marker genes enriched in the clusters, enabling identification of cell types from the liver immune cell experiment. Figure shows results projected in three dimensions using UMAP. B cells were identified by the expression *Cd19*, *Cd79a*, and *Cd79b*; Macrophages by *Cd14*, *Spi1*, and *Csf1r*; Mito High are cells with high expression of genes encoded by the mitochondrial genome; Neutrophils by *Mmp8* and *Ccl6*; NK cells by *Nkg7*; plasmacytoid Dendritic Cells (pDC) by *Siglech*; and T cells by *Cd3d*, *Cd3e*, and *Cd3g*. **(B)** Cells in the clusters with Macrophage markers (red, other cell types and colors dimmed) were selected for further analysis.

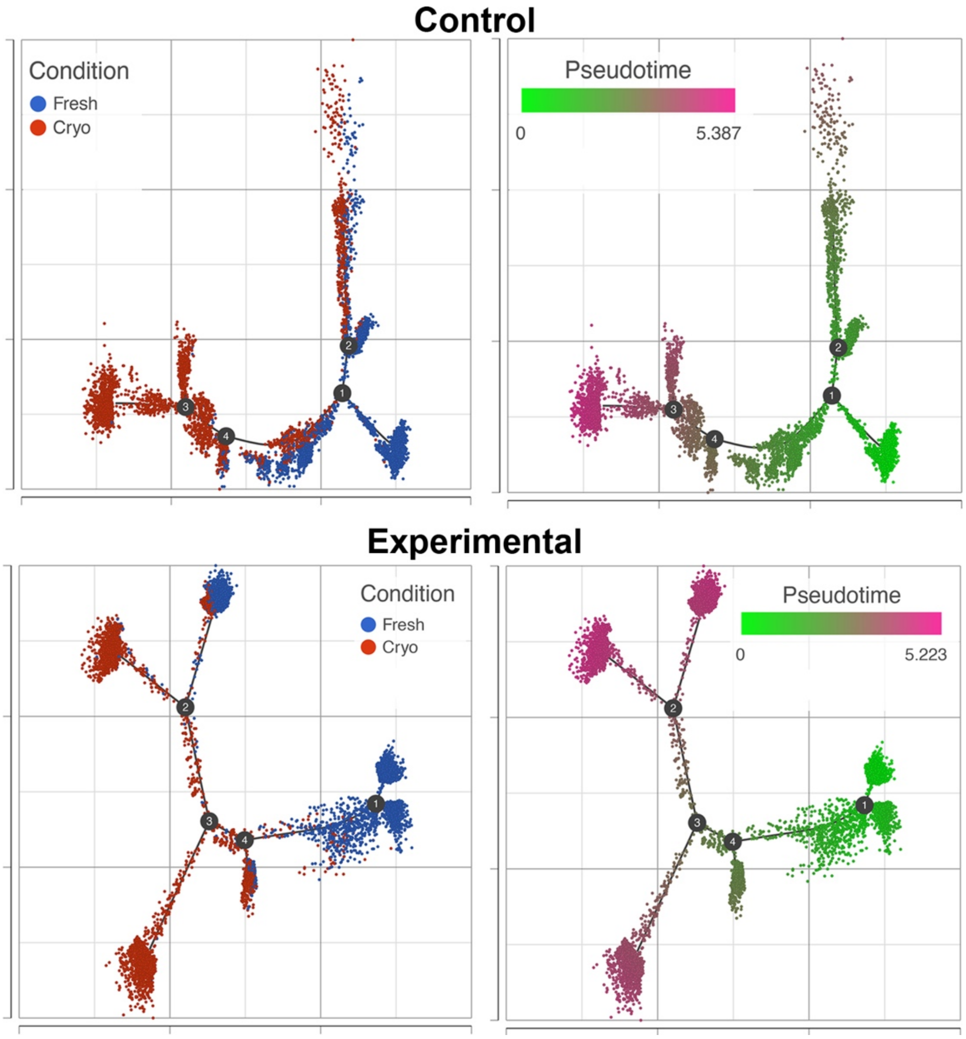

Figure S4. Trajectory analysis of fresh and cryopreserved rat liver macrophages (Related to Figures 5 and Results). Trajectory analysis for the two conditions of rat macrophage analyzed by scRNA-seq. The transition from fresh to the cryopreserved condition is indicated, as on the left, with pseudotime indicated on the right.

Transparent Methods
Experimental Model and Subject Details
Animals

Two male, five-year-old Indian origin rhesus macaques were purchased from New Iberia Research Center (New Iberia, LA). The monkeys tested negative for the indicated viral pathogens: SIV, SRV, STLV-1, B-virus, and measles; and bacterial pathogens: salmonella, shigella, campylobacter, yersinia, and vibrio. Macaques were housed in compliance with the Animal Welfare Act and the Guide for the Care and Use of Laboratory Animals in the nonhuman primate facilities at Department of Comparative Medicine, University of Nebraska Medical Center (UNMC). The primate facility at UNMC has been accredited by the AAALAC international. This study was reviewed and approved by the UNMC Institutional Animal Care and Use Committee under protocol 16-001-02-FC titled "Assessment of Antiretroviral Pharmacology in Lymphoid Tissues using the SIV macaque model" approved 2/25/2016. Animals were maintained in a temperature-controlled (23 ± 2° C) indoor climate with 12-hour light/dark cycle. They were fed Teklad Global 25% protein primate diet (#2055, Envigo) supplemented with fresh fruit or vegetables, and water ad libitum. The monkeys were observed twice daily for health status by the animal care and veterinary personnel.

At the start of study, both macaques were intravenously inoculated with 200 µl of a $SIV_{mac}251$ stock, which contained 2.48 x 10^9 copies/ml of SIV RNA. Virus stocks were provided by the Virus Characterization, Isolation and Production Core at Tulane National Primate Research Center (Covington, LA) as described previously (Del Prete et al., 2013). Five-weeks post-inoculation, combined antiretroviral therapy (cART) was initiated. One animal received cART consisting of two drugs: 4 mg/ml dolutegravir (DTG), 40 mg/ml emtricitabine (FTC) in a vehicle of 15% Kleptose and 0.1 N NaOH at a pH of 7.4, while the other was treated with four drug cART, consisting of 4 mg/ml DTG, 1mg/ml tenofovir alafenamide fumarate (TAF), 40 mg/ml FTC, 12 mg/ml maraviroc (MVC) in a vehicle of 15% Kleptose and 0.1 N NaOH at a pH of 7.4. Both animals were treated once daily with a subcutaneous injection of 1 ml cART/kg body weight.

Animals remained on the cART drug regimen for six months following suppression of virus in the plasma (viral load of <50 SIV copies/ml plasma) and were sacrificed as per experimental design for the above-named approved study (for animal 85T a total of 302 days of SIV infection, 277 days of cART, and documented suppression for 181 days, for 86T seven additional days for each measure). Plasma viral load was determined from EDTA-anticoagulated plasma separated from blood by centrifugation. Plasma was stored at -80° C until assessed for viral load. SIV RNA viral load was measured using a PCR-based assay by the Quantitative Molecular Diagnostics Core at the National Cancer Institute (Frederick, MD) essentially as described previously (Li et al., 2016). At necropsy, deeply anesthetized animals were perfused intracardially with sterile PBS containing 1 U/ml heparin to clear blood including blood-borne cells from the brain. Brains were harvested for histopathology and molecular studies, and approximately half of the brain then taken for single cell microglia/macrophages isolations and further experimental studies.

Isolation of total brain microglia/macrophages

Microglia/macrophages-enriched brain isolation was preformed using a modification of our previously described procedure (Marcondes et al., 2001). The brain was sectioned and meninges were removed in a 150 mm dish on ice with 4° C Hank's balanced salt solution (HBSS; Invitrogen, Carlsbad, CA). The remaining roughly minced tissue was homogenized with a Dounce homogenizer using additional 4° C HBSS as necessary. Homogenate was washed twice with 4° C HBSS and centrifuged at 4° C for 7 minutes at 550 x g. The brain tissue was then digested at in 37° C in HBSS containing 28 U/ml DNase I and 8 U/ml papain (approximately 2.5 U/gram of tissue) on a nutating mixing platform at 37° C for 30 minutes (Sigma, St. Louis, MO). While digesting, the tissue was triturated at 15 minutes to assist in dissociation. After digestion, the enzymes were inactivated by addition of 2.5% FBS (volume/volume). Digested tissue was centrifuged then resuspended in 25 mL of 4° C HBSS. The cell suspension was mixed with 7 mL of 90% Percoll (GE HealthCare, Pittsburg, PA) and centrifuged at 4° C for 15 minutes at 550 x g, with the acceleration set to 5 and the brake set to 1. The resulting fatty upper layer and HBSS were removed from the enriched microglia and macrophage pellet. The pellet was resuspended in 10 mL 4° C HBSS and passed through a 40 µm screen to remove cell clumps and/or aggregates. Cells were pelleted and, if necessary, resuspended in RBC lysis buffer for 3 minutes to eliminate any contaminating red blood cells. A final wash with 4° C HBSS was performed before the resulting cells were quantified on

both hemocytometer and Coulter Counter Z1.

The brain microglia/macrophages isolates were either used for scRNA-seq as described below, or cryopreserved for later use. To cryopreserve, cells were centrifuged at 4° C at 550 x g for 5 minutes and supernatant was removed. The pellet was dissociated by tapping and then resuspended by the dropwise addition of a solution of 4° C 10% DMSO in FBS at a concentration of 10 million cells per milliliter. Cells were transferred to cryopreservation tubes and placed in a Mr. Frosty for slow controlled freezing at -80° C. After 24 hours, cryotubes were transferred to liquid nitrogen for long-term storage.

scRNAseq preparation of fresh brain isolates

All procedures were completed using RNAase-free tubes and filter tips. Brain microglia/macrophages were washed in PBS, and stained with UV-blue live/dead assay for 30 minutes at 4° C. Cells were centrifuged at 300 x g for 8 minutes at 4° C and resuspended in e-bioscience flow cytometry staining buffer for 5 minutes at 4° C. Cells were again centrifuged at 300 x g for 8 minutes at 4° C and resuspended in MACS separation buffer with 0.1% BSA (Miltenyi, Gladbach, Germany). Cells were counted and volume was adjusted for staining with non-human primate CD11b microbeads (Miltenyi). Twenty million cells were reconstituted in 80 μL of MACS buffer and reacted with 40 μL of CD11b microbeads at 4° C for 15 minutes. After incubation, cells were washed with MACS buffer with 0.1% BSA, centrifuged at 300 x g for 8 minutes at 4° C, and the resulting pellet was resuspended in 1 ml of MACS buffer and loaded onto two MACS Separator LS columns. The positive CD11b cells were collected and isolates were counted on Coulter Counter Z1 and assessed for viability and concentration using a hemocytometer and trypan blue exclusion. CD11b-enriched isolates were then stained with Brilliant Violet 605-labeled anti-mouse/human CD11b antibody clone M1/70 (Biolegend, San Diego, CA) for 45 minutes at 4° C. Cells were washed with e-bioscience flow cytometry staining buffer and cell concentration was set to 5 million cells per ml for fluorescence activated cell sorting (FACS). Machine standards were set with unstained and single stained cells. Live/dead standards were also set using the Amine reactive compensation Bead kit (Invitrogen, Carlsbad, CA). Cells were sorted based on size, singlets, live, CD11b+ events using an Aria2 flow cytometer (BD Biosciences). Sorted cells were adjusted to a concentration of 1,000 cells per μl in DMEM/F12 with 10% FBS. FACS files were analyzed using FlowJo software (FlowJo, Ashland, OR).

scRNAseq preparation of cryopreserved brain isolates

Samples of cryopreserved enriched brain microglia/macrophages isolates, stored in liquid nitrogen described above, were rapidly thawed in a 37° C water-bath. Once thawed, 1 ml of media consisting of DMEM/F12 with 2.5mM L-glutamine and 15mM HEPES with 10% FBS, supplemented with 1% DNase (Sigma Aldrich) was slowly added to cells using filter tips. Cells were incubated at room temperature for 10 minutes, gently mixed at 5 minutes using a pipet. After 10 minutes, the cells were transferred dropwise into a 15 ml tube and 10 ml of the above media with 1% DNase containing media. Cells were incubated at room temperature for 5 minutes and centrifuged at 25° C 300 x g for 8 minutes and resuspended in 1 ml of DMEM/F12 with 20% FBS, and incubated at 37° C in a 5% CO_2 incubator for 15 minutes to enable recovery. Media was removed and cells were washed and counted using a Coulter Counter for use. Once cell concentration was known, cells were transferred to ice-cold PBS, and sequentially stained with the Live/Dead Assay, enriched immunomagnetically by positive selection by MACS using CD11b magnetic beads, and then stained with CD11b-BV605 and processed for FACS purification, all as described above for the fresh brain microglia/macrophage isolates.

scRNAseq capture and library preparation

Post-sort, fresh and cryopreserved isolates were concentrated to approximately 1,000 cells per μl, assessed by trypan blue for viability and concentration. Based on 10x Genomics parameters targeting 8,000 cells, the ideal volume of cells was loaded onto the 10x Genomics (Pleasanton, CA) Chromium GEM Chip and placed into Chromium Controller for cell capturing and library preparation. We used the 10x Genomics Single Cell 3' GEM, Library, & Gel bead kit v3. Briefly, the microfluidics combines the Single Cell 3' Gel Beads containing unique barcoded primers with a unique molecular identifier (UMI), followed by lysis of cells and barcoded reverse transcription of RNA, amplification of barcoded cDNA, fragmentation of cDNA (with peak consistently in the range of 470-500 bp), 5' adapter attachment and sample indexing as the manufacturer instructed with version 3 reagent kits. The prepared libraries were then sequenced using an Illumina Nextseq550 sequencer with the NSQ 500 hi- Output KT v2 (150 CYS)

(San Diego, CA).

Bioinformatic Analysis
Rhesus monkey samples

Bioinformatic analysis was performed basically as described in Niu et al., 2020. Demultiplexing and filtered feature barcode matrices generation was performed by Cell Ranger (version 3.1, 10x Genomics) pipeline. The raw base call (BCL) files were converted into FASTQ files, and the reads aligned to custom combined genomes of *Macaca mulatta* (Mmul 10) and the SIV genome (using NCBI reference sequence M33262.1, note no SIV sequences were identified). Cell Ranger also performed filtering, barcode counting, UMI counting, and sample aggregation. As a result, a gene expression matrix was generated containing the raw UMI counts for each cell for each sample, along with a matrix consisting of matrices of all samples.

Partek (St. Louis, MO, USA) Flow version 9.0 was used for in-depth analysis, with the gene expression matrices for each sample as the input. Single cell QA/QC was performed to filter out low-quality cells based on the Total UMI count (<800 or >15,000), Detected Gene count (<300 or >5,000), and Mitochondrial UMI proportion (>15%), resulting in the removal of 784 cells, leaving 23,792. This was followed by a noise reduction step, in which the genes that had zero expression in all cells were removed from further analysis. Normalization was performed by converting the UMI to CPM (counts per million reads), then adding 1 and converting to the $\log_2$ value.

Initial principle component analysis (PCA) was performed with a setting of 50 PCs for calculation. Graph-based clustering was performed using the first 15 PCs from the PCA (according to the PC scree plot), and Louvain as the clustering algorithm. Data visualization was performed using Uniform Manifold Approximation and Projection (UMAP). A cluster of contaminating lymphocytes was identified as described in the text, and those cells were excluded from further examination. Differentially expressed genes (DEGs) analysis to assess differences between the fresh and cryopreserved cells was performed using Gene Specific Analysis (GSA) in Partek Flow software. GSA functions to select the best statistic model for each gene's differential expression from its pool to produce p-values. The five response distributions in the pool are Normal, Lognormal, Lognormal with shrinkage, Negative Binomial, and Poisson distributions. Trajectory analysis and pseudotime calculation was performed (also using Partek Flow, using Monocle 2 (Qiu et al., 2017)) on the set of 538 DEGs, chosen with a fold-change of $> |1.5|$ and a false discovery rate of < 0.05 (Table S1).

Additional bioinformatic analyses were performed using human gene IDs of the rhesus monkey orthologs. Gene Set Enrichment Analysis (GSEA) (Subramanian et al., 2005) was performed on the expression data (using GSEA (version 4.1.0)). For the analysis, 1000 permutations were used to calculate the p values with permutation type set to phenotype. To prepare the expression data, the cellranger aggr function of Cell Ranger was used to aggregate the matrices of all samples, followed by Loupe Browser (version 4.2.0, 10x Genomics) to calculate the average UMI for each gene per sample considering the size factor. The source of the gene sets are referenced within the text, the Gene Ontology (GO) lists were obtained from the Molecular Signatures Database (Liberzon et al., 2015). To facilitate pathway analysis the gene set was filtered to include a curated list containing only rhesus monkey genes with human homologues, and then removing non-protein coding genes, such as ribosomal RNA, miRNA, snRNA, lncRNA, as well as genes that coded for ribosomal proteins and mitochondrial-encoded proteins, as described (Niu et al., 2020). A further noise reduction step was taken to exclude genes that were not expressed in at least 99.9% of the remaining cells. Ingenuity Pathway Analysis (IPA) (QIAGEN, Germantown, MD, USA) was used to examine canonical pathway alterations. Metascape (Zhou et al., 2019) was used to perform interactome analysis using the molecular complex detection (MCODE) clustering algorithm (Bader and Hogue, 2003)..

Rat immune cell data

A total of eight rat immune scRNA-seq samples (Wohnhaas et al., 2019) were downloaded from GSE127248 in the MEX format. Sample data contain the unfiltered feature-barcode matrices of all barcodes. The data were uploaded to Partek Flow, and cell barcode QA/QC was performed to determine whether a given cell barcode is associated with a cell for all 8 samples, followed by leaving a total of 35,349 cells for further analysis. Single cell QA/QC was then performed for Total UMI count and Detected Gene count, leaving a total of 31,604 cells. Noise reduction filtering was used to exclude genes that were not expressed in at least 99.9% of the remaining cells, followed by PCA, graph-based clustering, and

visualization using UMAP. Clusters containing macrophages (as shown in Figure S3) were selected, resulting in 10,405 cells. DEGs analysis, trajectory analysis and pseudotime calculation were performed similarly to the above analysis of the rhesus monkey samples.

Statistics

Alpha was set to < 0.05 for all analyses reported. Differentially expressed genes were determined using gene-specific analysis as performed by Partek Flow version 9.0. Pearson correlation analysis was performed using Prism version 9.0.0 (GraphPad Software, San Diego, CA).

KEY RESOURCES TABLE

REAGENT or RESOURCE	SOURCE	IDENTIFIER
Antibodies		
Anti-CD11b antibody clone M1/70, BV605	BioLegend	Cat# 101257
CD11b microbeads, non-human primate	Miltenyi Biotec	Cat# 130-091-100
Bacterial and Virus Strains		
$SIV_{mac}251$	Tulane National Primate Research Center	Del Prete et al., 2013
Biological Samples		
Indian origin rhesus macaques	New Iberia Research Center	N/A
Chemicals, Peptides, and Recombinant Proteins		
Kleptose HPB Parenteral GradeEP-USP/NF	Roquette	Cat# 346111107
Dolutegravir Sodium	Adooq Biosciences	Cat# A16162
Maraviroc	Adooq Biosciences	Cat# A10556
Emtricitabine	Gilead Biosciences	N/A
Tenofovir alafenamide	Gilead Biosciences	N/A
DNase I (for cryopreserved cell recovery)	Sigma Aldrich	Cat# 10104159001
DNase (for microglia isolation from brain)	Sigma Aldrich	Cat# D4527-200KU
Papain	Sigma Aldrich	Cat# P5306
DMSO	Sigma Aldrich	Cat# D2650
Critical Commercial Assays		
Live Dead assay UV blue	Invitrogen	Cat# L23105
ArC Amine reactive compensation Bead kit	Invitrogen	Cat# A10346
Single Cell 3' GEM, Library, & Gel bead kit v3	10x Genomics	Cat# 1000075
Single Cell 3' Chip B Kit	10x Genomics	Cat# 1000074
NSQ 500 hi- Output KT v2 (150 CYS)	Illumina	Cat# FC-404-2002
NSQ 500 hi- Output RGT CART v2 (150 CYS)	Illumina	Cat# 15057931
NSQ 500 hi- Output FC CART v2	Illumina	Cat# 15065973
Deposited Data		
NCBI GEO database, accession # GSE162663	This paper	https://www.ncbi.nlm.nih.gov/geo/query/acc.cgi?acc=GSE162663
NCBI GEO database, accession # GSE127248 (rat liver immune cells)	Wohnhass, CT, et al., Sci Rep. 2019 9:10699.	https://www.ncbi.nlm.nih.gov/geo/query/acc.cgi?acc=GSE127248

NCBI GEO database, accession # GSE160384 (monkey microglia)	Niu, M, et al., Viruses 2020, 12:1297.	https://www.ncbi.nlm.nih.gov/geo/query/acc.cgi?acc=GSE160384
Macaca mulatta genome (Mmul_10)	Genome Institute at Washington University	https://uswest.ensembl.org/Macaca_mulatta/Info/Index
SIVmac239 complete proviral genome	National Center for Biotechnology Innovation	https://www.ncbi.nlm.nih.gov/nuccore/M33262
Software and Algorithms		
Cell Ranger version 3.1	10x Genomics	https://support.10xgenomics.com/single-cell-gene-expression/software/pipelines/latest/what-is-cell-ranger
Loupe Browser version 4.2.0	10x Genomics	https://support.10xgenomics.com/single-cell-gene-expression/software/visualization/latest/installation
Partek Flow version 9.0	Partek	https://www.partek.com/partek-flow
Prism version 9.0.0	GraphPad Software	https://www.graphpad.com
Metascape update 09-16-2020	Zhou Y, et al., Nature Communication 2019, 10:1523	https://metascape.org/gp/index.html#/main
GSEA version 4.1.0	Subramanian A, et al, PNAS 2005, 102:15545	https://www.gsea-msigdb.org/gsea/msigdb/index.jsp
Molecular Signatures Database version 7.2	Subramanian A, et al, PNAS 2005, 102:15545	http://www.gsea-msigdb.org/gsea/msigdb/index.jsp

Supplemental References

Bader, G.D., and Hogue, C.W. (2003). An automated method for finding molecular complexes in large protein interaction networks. BMC Bioinformatics *4*, 2.

Del Prete, G.Q., Scarlotta, M., Newman, L., Reid, C., Parodi, L.M., Roser, J.D., Oswald, K., Marx, P.A., Miller, C.J., Desrosiers, R.C., *et al.* (2013). Comparative characterization of transfection- and infection-derived simian immunodeficiency virus challenge stocks for in vivo nonhuman primate studies. J Virol *87*, 4584-4595.

Li, H., Wang, S., Kong, R., Ding, W., Lee, F.H., Parker, Z., Kim, E., Learn, G.H., Hahn, P., Policicchio, B., *et al.* (2016). Envelope residue 375 substitutions in simian-human immunodeficiency viruses enhance CD4 binding and replication in rhesus macaques. Proc Natl Acad Sci U S A *113*, E3413-3422.

Liberzon, A., Birger, C., Thorvaldsdottir, H., Ghandi, M., Mesirov, J.P., and Tamayo, P. (2015). The Molecular Signatures Database (MSigDB) hallmark gene set collection. Cell Syst *1*, 417-425.

Qiu, X., Mao, Q., Tang, Y., Wang, L., Chawla, R., Pliner, H.A., and Trapnell, C. (2017). Reversed graph embedding resolves complex single-cell trajectories. Nat Methods *14*, 979-982.

Subramanian, A., Tamayo, P., Mootha, V.K., Mukherjee, S., Ebert, B.L., Gillette, M.A., Paulovich, A., Pomeroy, S.L., Golub, T.R., Lander, E.S., *et al.* (2005). Gene set enrichment analysis: a knowledge-based approach for interpreting genome-wide expression profiles. Proc Natl Acad Sci U S A *102*, 15545-15550.

Wohnhaas, C.T., Leparc, G.G., Fernandez-Albert, F., Kind, D., Gantner, F., Viollet, C., Hildebrandt, T., and Baum, P. (2019). DMSO cryopreservation is the method of choice to preserve cells for droplet-based single-cell RNA sequencing. Sci Rep *9*, 10699.

Zhou, Y., Zhou, B., Pache, L., Chang, M., Khodabakhshi, A.H., Tanaseichuk, O., Benner, C., and Chanda, S.K. (2019). Metascape provides a biologist-oriented resource for the analysis of systems-level datasets. Nat Commun *10*, 1523.
